# An Updated Comprehensive Pharmacovigilance Study of Drug- Induced Breast Cancer Based on FDA Adverse Event Reporting System Data

**DOI:** 10.2174/0109298673376219251029174252

**Published:** 2026-01-07

**Authors:** Ziran Qiu, Xinyu Liu, Wenqing Cao, Rui Li, Jun Yang, Chengyu Wang, Zhong Li, Xiaoqin Yao, Yuan Chen, Chunhua Ye, Shanzheng Chen, Na Jin

**Affiliations:** 1 Department of Breast and Thyroid Surgery, Loudi Central Hospital, Loudi, 417000, China;; 2 Department of Otolaryngology Head and Neck Surgery, Loudi Central Hospital, Loudi, 417000, China

**Keywords:** Breast cancer, drug safety, adverse events, precision medicine, FAERS database, reporting Odds ratio (ROR)

## Abstract

**Introduction:**

To address critical knowledge gaps in understanding drug-specific risk profiles of breast cancer (BC), ultimately informing regulatory decision-making and clinical practice.

**Methods:**

This study conducted a retrospective drug safety study based on the FDA's Adverse Event Reporting System (FAERS) database, examining BC drug treatment data from 2004 to 2024. The research involved data cleaning, standardization of drug names, and the application of statistical analysis methods such as the proportional imbalance method and the Reporting Odds Ratio (ROR) to assess potential associations between drugs and adverse events.

**Results:**

Significant differences were determined in adverse reaction reports among different drugs in BC treatment. Specific drugs such as trastuzumab, lapatinib, and certain endocrine therapy medications accounted for a higher proportion of adverse event reports and exhibited higher ROR values. Additionally, the study identified that cardiotoxicity, osteoporosis, ovarian function impairment, and immune complications are adverse reactions requiring special attention in BC drug treatment.

**Discussion:**

This comprehensive analysis of FAERS data highlights the significance in drug-induced adverse event reporting across BC therapies. This work promotes the advancement of patient safety by providing actionable evidence for therapeutic decision making in the oncology of BC. A limitation of this study is the lack of integration of potential mechanisms for adverse events in BC patients.

**Conclusion:**

Targeted therapies of lapatinib, fulvestrant, and endocrine agents (letrozole or ribociclib) show disproportionately high AE reporting odds ratios, necessitating vigilant monitoring. HER2-positive BC treatments (trastuzumab) and TNBC agents (sonidegib) exhibit distinct risk profiles, advocating for personalized risk-benefit assessments for BC patients.

## 1 INTRODUCTION

Pursuant to the data from the American Cancer Society, it is estimated that there will be 1,918,030 new cancer cases and 609,360 cancer deaths in 2022 [1]. Breast cancer (BC) has long been the leading cause of malignancy among women, occupying a significant position in the female mortality rate [2]. Over the period from 2014 to 2018, the annual incidence rate of female BC showed a slight upward trend of 0.5%. Concurrently, since 2011, the annual growth rate of advanced BC has remained in the range of 4% to 6%. Additionally, the proportion of patients diagnosed with advanced BC has also increased, rising from 3.9% to 8.2% [1].

The classification of BC is based on the status of hormone receptors (including estrogen receptor and progesterone receptor) expressed by the tumor, as well as the status of the human epidermal growth factor receptor 2 (HER2). The varying expression levels of these biomarkers categorize BC into multiple subtypes, each associated with a specific treatment regimen. With the continuous advancement of precision medicine technologies, the treatment of BC is progressively shifting towards a personalized and stratified treatment strategy, aiming to achieve a more precise disease management for each patient [3]. The integrated application of local treatment modalities, including surgery and radiation therapy, along with systemic treatments encompassing endocrine therapy, chemotherapy, targeted therapy, and immunotherapy, offers early-stage BC patients the potential and hope for a cure [4, 5].

Over the past 10 years, the field of BC pharmacotherapy has undergone significant innovations, particularly in molecular targeting and personalized medicine. Current treatment strategies are gradually shifting from the traditional one-size-fits-all approach to precision medicine based on tumor molecular characteristics, such as utilizing the status of hormone receptors and HER2 to tailor treatment regimens [6]. Novel targeted agents, like Trastuzumab for HER2-positive BC, have significantly improved patient survival rates. Simultaneously, gene expression analysis tools like Oncotype DX and MammaPrint provide robust support in assessing recurrence risk and guiding therapy [7]. While immunotherapy and anti-angiogenic therapy are still in early stages of research, they have demonstrated potential in BC treatment [8, 9]. A recent real-world analysis indicated the safety and efficacy of sacituzumab govitecan in metastatic triple-negative breast cancer patients [10]. Data supporting immunotherapy in the metastatic triple-negative breast cancer setting are not concordant, but there have been some positive phase III trials, including IMpassion130 and KEYNOTE-355 [11]. In addition, a superior risk of serious adverse events in patients receiving chemoimmunotherapy was determined compared to those only given chemotherapy [12]. Immune checkpoint inhibitors (ICIs) were associated with different immune-related adverse events [13]. In order to find a non-invasive prognostic marker for cancer, it has been demonstrated that an elevated neutrophil-to-eosinophil ratio (NER) has an association with poorer survival time [14], suggesting its potential utility in immune-related adverse events. These advancements underscore the importance of pharmacovigilance to ensure the safety of increasingly complex therapeutic regimens.

With the growing complexity of pharmaceutical therapies and prolonged drug exposure in clinical practice, unintended carcinogenic effects remain underrecognized, particularly in post-marketing surveillance. Traditional drug approval processes, reliant heavily on preclinical and short-term clinical trials, often fail to capture delayed or rare adverse events. The research progress in adverse reactions to BC pharmacotherapy primarily focuses on four aspects: cardiotoxicity, osteoporosis, ovarian function impairment, and immune complications. Radiotherapy and chemotherapy may increase the risk of heart disease, especially when combined, with cardiac fibrosis playing a significant role in radiation-chemotherapy-induced cardiac injury [15]. Additionally, these treatments can lead to osteoporosis, likely due to chemotherapy-induced ovarian failure and calcium reduction [15]. The damage to the ovaries caused by chemotherapy may also affect reproductive health and quality of life, as evidenced in animal models. With the widespread application of immune checkpoint inhibitors, predicting and treating immune complications has become increasingly crucial, as these drugs, while effective, can also induce a series of immune-related adverse reactions that require rigorous clinical monitoring during treatment [16, 17]. Pharmacovigilance transforms passive drug monitoring into a proactive system that bridges gaps between clinical research and real-world practice [18]. Understanding these adverse events is essential for developing new strategies to minimize side effects and improve patients' quality of life. By ensuring continuous evaluation of drug benefits *versus* risks, it ultimately empowers healthcare providers to deliver safer, more effective therapies.

Adverse drug reactions affect the pathological process of breast cancer and its complications in many ways, such as direct tissue injury, tumor biological changes, and treatment interruption. Several breast cancer patients discontinue therapy due to drug-induced adverse events, increasing recurrence risks and costs [19]. Given the widespread use of anti-cancer drugs for BC patients, exploring the association between these medications and adverse events has become paramount. The Food and Drug Administration's (FDA) Adverse Event Reporting System (FAERS), as a global spontaneous reporting mechanism, amasses significant real-world data. FAERS has been extensively utilized to detect risk signals for adverse events. The objective of this study is to assess the potential associations between various anti-BC drugs and adverse events by analyzing standardized data from the FAERS database. This comprehensive pharmacovigilance study aims to address critical knowledge gaps in understanding the epidemiology and drug-specific risk profiles associated with BC, ultimately informing regulatory decision- making and clinical practice.

## 2 METHODS

### 2.1 Data Sources

We conducted a retrospective drug safety study based on the FAERS database, an internationally recognized and publicly accessible repository for adverse event reports. This database consolidates voluntary reports from diverse sources such as healthcare professionals, patients, pharmacists, and pharmaceutical manufacturers, providing crucial support to the FDA's postmarket surveillance of drugs and biological products. FAERS encompasses a wide range of information, including patient demographics, detailed adverse event descriptions, medication records, reporting sources, treatment durations, drug indications, and patient outcomes. It adheres to international safety reporting standards established by the International Conference on Harmonization, where all adverse events are coded using preferred terms (PTs) from the Medical Dictionary for Regulatory Activities. These PTs enable a comprehensive description of various medical phenomena, including clinical manifestations, symptoms, diagnoses, and laboratory test results, medical and family histories, among others. Furthermore, PTs can be categorized into higher-level group terms (HLGTs) and system organ classes (SOCs) or grouped using Standardized MedDRA Queries (SMQs) for specific medical conditions.

The FAERS data files encompass seven types of data documents: DEMO (demographic and administrative information), DRUG (drug information), REAC (coded adverse events), OUTC (patient outcomes), RPSR (report sources), THER (therapy start and end dates for reported drugs), and INDI (indications for drug administration). Within the FAERS database architecture, these files are interconnected through specific identification numbers such as PRIMARYID. The database undergoes quarterly updates, and all data is accessible *via* https://fis.fda.gov/extensions/FPD-QDE-FAERS/FPD-QDE-FAERS.html. To ensure the inclusion of the most recent reports, we extracted all FAERS reports recorded from the first quarter of 2004 up to the present date.

### 2.2 Data Cleaning

Given that the database collects data through spontaneous reporting, there may be duplicate reports or those that have been withdrawn/deleted. Therefore, the FDA's official guidance document provides rules for de-duplication and lists of reports that need to be deleted. This study strictly followed the FDA's official guidance document for data cleaning.

The data cleaning rules were as follows: Firstly, to remove duplicate reports, we selected records based on the PRIMARYID, CASEID, and FDA_DT fields from the DEMO table, following the FDA's recommended method. Reports with the same CASEID were sorted by FDA_DT, and the one with the latest FDA_DT was retained. For those with identical CASEID and FDA_DT, the report with the highest PRIMARYID was kept. Secondly, since the first quarter of 2019, each quarterly data package has included a list of deleted reports. After de-duplication, we further excluded reports based on their CASEIDs listed in the deleted reports list.

Adverse events were included if they met MedDRA Preferred Term (PT) definitions and occurred in ≥3 reports for a given drug. Events with incomplete causality assessments or missing critical fields (*e.g.*, drug name, outcome) were excluded. Limitations of data cleaning include potential underreporting of mild adverse events and selection bias favoring severe or novel reactions. To mitigate this, we applied disproportionality analysis adjusted for reporting trends over time.

Inclusion criteria: (1) Primary Breast Cancer Therapies: Anticancer drugs approved for breast cancer, including chemotherapy, hormonal therapies, targeted agents, and immunotherapies; (2) Concomitant/Supportive Care Drugs: Medications reported in FAERS alongside breast cancer treatment, even if not breast-cancer-specific, to capture real-world polypharmacy risks; (3) Off-Label or Investigational Uses: Drugs reported for use in breast cancer patients, even if their primary indication is unrelated.

Exclusion criteria: (1) Drugs with No Recorded Association with Breast Cancer Patients: Agents exclusively used for unrelated conditions (*e.g.*, oral contraceptives not linked to breast cancer treatment, over-the-counter drugs without reported oncology use), unless flagged by proportionality analysis as potential signal generators; (2) Duplicate Entries: Generic vs. brand-name drugs (*e.g.*, “adalimumab” and “Humira” merged to avoid double-counting); (3) Non-Pharmacological Interventions: Devices, biologics not classified as drugs, or dietary supplements (unless explicitly labeled as such in FAERS).

### 2.3 Utilization of MedDRA Dictionary

The current latest version of the Medical Dictionary for Regulatory Activities (MedDRA) dictionary (MedDRA 27.0) was applied to encode the adverse event terms in the FAERS database.

Since the adverse event terms in the FAERS database are described using preferred terms (PTs) from the MedDRA, and considering that the MedDRA dictionary undergoes updates in March and September of each year, which involve adjustments to the PT hierarchy and corresponding changes to the System Organ Class (SOC) categories, it was necessary to re-calibrate the PT names in the FAERS database using the current latest version of the MedDRA dictionary. Furthermore, the updated SOC categories and PTs from the latest MedDRA dictionary were obtained for subsequent analysis.

Subtype-specific cases (HER2+, TNBC) were identified *via* structured fields (when available) and NLP of free-text sections, requiring explicit mention of subtype terminology (*e.g.*, 'HER2-positive,' 'triple-negative'). Reports lacking clear subtype documentation were classified as 'breast cancer, unspecified'.

### 2.4 Analysis Methods

SAS 9.4 was employed for statistical analysis, as it is one of the tools recommended by the FDA for mining the FAERS database.

This study utilized the proportional imbalance method for signal detection of adverse drug events. The following table illustrates the composition of the four-table structure employed in the proportional imbalance analysis (Tables **1**, **2**):

### 2.5 Data Elucidation

To enable batch calculation of signals for all drugs and extraction of specific adverse event signals associated with each drug at the database level, it was imperative to standardize all drug names recorded in the database. This study employed multiple strategies to achieve drug name standardization. Specifically, the identification and matching of drug names in the database were initially conducted with reference to the WHO DRUG dictionary, a comprehensive database that encompasses over 600,000 distinct trade names. For those drug names that remained unidentified, the Medex_UIMA tool was further applied for recognition. However, given the inherent limitations of Medex_UIMA in drug name recognition, multiple iterations were necessary to optimize the results. Despite these efforts, some drug names could not be fully automated for identification. Consequently, for those unidentified names with frequencies exceeding 50, manual standardization was undertaken.

Despite the significant efforts invested in the standardization process of drug names, we acknowledge that achieving 100% accuracy is not feasible. For drug names recorded after 2014, we estimate the recognition accuracy to exceed 99%. In contrast, for drug names before 2014, the anticipated accuracy ranges between 90% and 95%. While complete precision in standardization was not achieved, this minor discrepancy in accuracy has a negligible impact on the overall results and trends derived from database mining.

### 2.6 Statistics

Data processing and evaluation were conducted using SAS software (version 9.4). Differences in values among multiple groups were assessed using the Kruskal-Wallis test and Dunn’s test. All odds ratios were adjusted based on patients having complete information for each of these covariables (ie, women missing data for one or more of these covariables were dropped from the regression analysis). All statistical tests were two-sided.

## 3 RESULTS

### 3.1 Data Filtering Flowchart

We downloaded adverse event report data from the official website of the US Food and Drug Administration (FDA), spanning from the first quarter of 2004 to the first quarter of 2024, encompassing a total of 81 quarters of information. The initial total number of reports obtained amounted to 21,161,817 cases. We excluded 3,534,477 duplicate reports and then conducted thorough data cleaning, ultimately retaining 17,627,340 reports for analysis. Among these 17,627,340 reports, a total of 153,287 cases reported specific adverse events. The total number of adverse events recorded in these reports was 52,373,206 occurrences, with 155,269 occurrences specifically related to the adverse events of interest. Please refer to (Fig. **1**) for a detailed flowchart. The PT terms related to specific adverse events are presented in Table **S1**.

### 3.2 Clinical Characteristics of BC Patients

From the first quarter of 2004 to the first quarter of 2024, the FAERS database documented adverse drug reactions in 88,433 patients with BC. The clinical characteristics of these patients are detailed in Table **3**. In Fig. (**2**), the proportions of BC Patients with Specific Adverse Events were divided based on (a) Gender, (b) Age, (c) OCCP, (d) Get Data Year, (e) Serious Report or not, (f) Time of Adverse Event Occurrence, (g) Outcomes of death, life-threatening, hospitalization, disability, congenital anomaly, required intervention, and other serious outcomes, (h) Time to Event. The majority of the patients were female, accounting for 88.4% of the total, with the principal age range being 45 to 64 years old. The year with the highest number of reports was 2021, representing 42.99% of all reports, followed by 2022 with 11.76%. The median age of the entire patient population was approximately 58 years. Consumers constituted the largest proportion of reporters, at 39.13%. The United States had the highest proportion of reports among countries, with 64.10%. The proportion of serious reports is 99.33%. Regarding the outcomes of adverse events, the primary outcome was hospitalization, followed by death and events that were life-threatening (Fig. **2**).

### 3.3 Spectrum of Specific Adverse Reactions at the PT Level in BC Patients

In the domain of BC therapy, a ranking of the top 50 medications based on the frequency of reports for AEs is delineated in Table **4**. Commonly utilized targeted therapies, such as trastuzumab, have yielded 270 reported cases of AEs with a ROR of 1.72, accompanied by a 95% CI ranging from 1.52 to 1.93. Capecitabine, a widely implemented chemotherapeutic agent, accounts for 286 reported AEs with an ROR of 1.09 and a 95% CI from 0.97 to 1.22. Lapatinib, a tyrosine kinase inhibitor, has been associated with 202 reported cases of specific AEs, boasting an ROR of 3.40 with a 95% CI between 2.96 and 3.91. Within the class of aromatase inhibitors, letrozole has been linked to 700 reported cases of AEs, with an ROR of 5.52 and a 95% CI of 5.12 to 5.94. Anastrozole has seen 295 reported AEs with an ROR of 3.28 and a 95% CI from 2.92 to 3.68. Exemestane has resulted in 194 reported cases of specific AEs, featuring an ROR of 5.11 and a 95% CI of 4.44 to 5.89. Fulvestrant has garnered 249 reported AEs, with an ROR of 4.19 and a 95% CI from 3.70 to 4.75. Additionally, the selective estrogen receptor modulator, raloxifene, has been associated with 248 reported AEs, demonstrating an ROR of 7.33 with a 95% CI of 6.47 to 8.31. These data provide a comprehensive insight into the safety profile of medications utilized in BC treatment, offering vital information for clinical decision-making and patient consultation. Table **5** and Table **S2** presents the top 50 drugs ranked by the frequency of specific adverse events occurring in BC patients.

### 3.4 Characteristics of Metastatic BC Patients

The FAERS database encompassed a total of 9,254 patients with metastatic BC who experienced adverse events post-treatment. The clinical characteristics of these patients are presented in Table **6**. Among them, 8,597 (92.90%) were female, primarily within the age range of 45 to 64 years. The median age of the entire cohort was approximately 59 years. In terms of temporal distribution, the highest proportion of reports was from 2008, accounting for 9.83%, followed by 2023 with 9.30%. Geographically, the United States had the highest proportion of reports, at 21.55%. Notably, severe adverse events constituted a significant majority of the reports, accounting for 97.40% of the total. Regarding the outcomes of these adverse events, death was the primary consequence, followed by hospitalization and life-threatening situations.

### 3.5 Spectrum of Specific Adverse Reactions at the PT Level in Metastatic BC Patients

Among targeted therapies, 378 specific adverse events (SAEs) related to trastuzumab were reported, with a reporting odds ratio (ROR) of 23.92 (95% CI: 21.58-26.52); 215 SAEs associated with lapatinib were reported, with an ROR of 35.40 (95% CI: 30.91- 40.55); 37 SAEs specific to neratinib were reported, yielding an ROR of 30.34 (95% CI: 21.95-41.93). Additionally, 32 SAEs related to pertuzumab were reported, with an ROR of 10.65 (95% CI: 7.53-15.08).

In endocrine therapies, 668 SAEs specific to letrozole were reported, exhibiting an ROR of 53.84 (95% CI: 49.75-58.27); 204 SAEs associated with anastrozole were reported, with an ROR of 22.07 (95% CI: 19.21-25.36); 137 SAEs specific to exemestane were reported, yielding an ROR of 34.88 (95% CI: 29.45-41.32); and 610 SAEs related to ribociclib were reported, with an ROR of 55.07 (95% CI: 50.72-59.81).

Within chemotherapeutics, 319 SAEs specific to capecitabine were reported, demonstrating an ROR of 11.97 (95% CI: 10.71-13.39); 73 SAEs associated with docetaxel were reported, with an ROR of 2.37 (95% CI: 1.88-2.99). Detailed reports of SAEs related to other medications are presented in Table **7**.

### 3.6 Clinical Features of HER2-Positive BC Patients

Among 318 HER2-positive BC patients who experienced adverse reactions post-treatment, the clinical characteristics are detailed in Table **8**. The majority of patients were female, accounting for 78.93% (n=251), primarily concentrated within the age range of 45 to 64 years, with an overall median age of approximately 54 years. In terms of temporal distribution, the highest proportion of cases were reported in 2023, accounting for 17.30%, followed by 2021 with 14.78%. Geographically, the highest proportion of patients was reported from the United States, reaching 26.73%. Regarding the severity of adverse events, 96.54% of the reports were classified as serious. As for the outcomes of adverse events, hospitalization was the most prevalent, followed by life-threatening situations and fatal cases.

### 3.7 Range of Specific Adverse Reactions at the PT Level in HER2-Positive BC Patients

Table **9** and Table **S3** summarizes the key findings from ranking the top 50 drugs based on the frequency of specific adverse event reports in HER2-positive BC. Trastuzumab, a monoclonal antibody used in the treatment of HER2-positive cancers, reported the highest number of cases with a ROR of 221.11, indicating a strong association with the specified adverse events. Other targeted therapies, such as pertuzumab and trastuzumab emtansine, also exhibited elevated RORs, signifying a notable risk profile in the treatment of HER2-positive BC. Hormonal therapeutic agents, including anastrozole and letrozole, demonstrated moderate RORs, reflecting their utilization in the management of hormone receptor-positive BC. Docetaxel, a chemotherapeutic agent, featured a ROR that underscores its frequent use across various stages of BC treatment.

### 3.8 Fundamental Clinical Data on Triple-Negative BC Patients

Table **10** presents a summary of the basic information regarding specific adverse events in patients with triple-negative BC (TNBC). Among the 303 cases reported, females accounted for 92.11%, with the majority of patients aged 45-64 years (31.91%), followed by those over 65 years (11.84%). The average age was 52.48 years, and the reporting years were concentrated between 2019 and 2022. The reporters were primarily healthcare professionals (38.16%) and consumers (36.84%). The United States was the primary country of occurrence, accounting for 44.08%, followed by Germany at 10.20%. The reporting countries were primarily located in North America (52.30%). The severity of adverse events was extremely high, with 99.67% being classified as serious, including a fatality rate of 10.86%. The onset of adverse events mostly occurred within 0-30 days after drug administration (5.26%), but 81.91% of the data was missing or contained abnormal values.

### 3.9 Spectrum of Specific Adverse Reactions at the PT Level in Triple-Negative BC Patients

Table **11** and Table **S4** provides a frequency ranking of drugs associated with specific adverse events in triple-negative BC (TNBC) patients, listing the top 50 drugs. Among these, Atezolizumab led with 22 reports, exhibiting a high ROR of 85.05, indicating a strong association with adverse events. Sacituzumab Govitecan followed closely with 15 reports and an ROR of 286.78, demonstrating an exceptionally high ratio that may signify potential risks in TNBC treatment.

Other notable drugs include Docetaxel and Capecitabine, with 11 and 10 reports respectively, and RORs of 11.20 and 11.38, both showing strong associations with adverse events. Denosumab and Sonidegib appeared on the list with 9 and 8 reports, respectively, and RORs of 5.06 and 885.20, the latter of which is particularly striking. Sonidegib blocks the Hedgehog signaling pathway by inhibiting the SMO protein, which plays a key role in embryonic development and tissue homeostasis, and is particularly active in tissues such as skin, muscle, gastrointestinal tract, and bone. In TNBC treatment, inhibition of this pathway may interfere with the repair and regeneration of normal tissues, resulting in the adverse effects [20].

The table also encompasses hormonal and immunotherapy agents such as Fulvestrant and Etanercept, whose use in TNBC treatment may be linked to adverse events experienced by patients. Additionally, chemotherapeutic drugs like Cyclophosphamide and Methotrexate are present, likely due to their known side effects. Moreover, certain drugs with fewer reports but notable RORs, like Olaparib and Risankizumab, also warrant attention.

### 3.10 Basic Clinical Profile of Hormone Receptor Positive BC Patients

Table **12** comprehensively presents the basic profile of specific adverse events experienced by patients with hormone receptor-positive BC. Among the 295 reported cases, female patients accounted for the vast majority (87.16%), while male patients had a lower proportion (5.74%), and 7.09% of cases did not specify gender information. In terms of age composition, middle-aged patients (45-64 years old) accounted for the highest proportion (34.80%), followed by elderly patients (65 years and above) with 18.92%. In contrast, young patients (18-44 years old) accounted for a relatively low proportion of only 10.47%. The average age of patients was 56.59 years, with a median age of 56 years.

The distribution of report years revealed that 2021 (15.20%) and 2023 (18.24%) were the years with a relatively concentrated number of reports, indicating a significant growth trend in the number of adverse event reports in recent years. The main sources of reports were physicians (35.47%) and consumers (35.14%), reflecting a possible increase in patients' self-protection awareness and the transparency of medical information.

Geographically, North America (46.96%) and Europe (32.09%) were the two regions with the highest number of reports, with the United States accounting for 39.86% as the most concentrated country. Regarding the severity of the reports, the majority (97.64%) were classified as serious, with life-threatening cases accounting for 7.43%, hospitalizations accounting for 25.00%, and fatal cases representing a relatively low proportion of 0.68%.

Regarding the occurrence time of adverse events, most reports (82.77%) had missing or abnormal data, while the specific time points were concentrated mostly after 360 days of medication (11.82%). The average and median occurrence time of adverse events were 1233.10 days and 892 days, respectively, indicating that some patients experienced adverse events after a relatively long period, posing higher requirements for the safety and monitoring of long-term treatment.

### 3.11 Range of Specific Adverse Reactions at the PT Level in Hormone Receptor-Positive BC Patients

Table **13** and Table **S5** provides a frequency ranking of drugs associated with specific adverse events in patients with hormone receptor-positive BC, listing the top 50 drugs. Among these drugs, Levonorgestrel emerged as the leading drug with 29 reported cases, boasting a significantly higher ROR of 12.82, indicating a strong association with adverse events. Closely following was Letrozole with 20 reported cases and an exceptionally high ROR of 49.73, exhibiting a strong pharmacovigilance signal.

Estradiol and Anastrozole ranked next with 15 and 10 reported cases, respectively, carrying RORs of 27.12 and 34.12. The high ROR values of these hormonal agents may be attributed to their widespread use in BC treatment. Notably, Ranitidine, though not a BC treatment drug, also appeared on the list with 10 reported cases and an ROR of 3.15 under concomitant/supportive therapy.

It is worth mentioning that drugs such as Everolimus and Ocrelizumab, despite having fewer reported cases, exhibited high RORs of 11.10 and 8.91, respectively, suggesting the need for particular attention to their safety in BC treatment. Fulvestrant and Tamsulosin also displayed high RORs of 35.76 and 32.66. The adverse reactions of Fulvestrant mainly result from the estrogen deprivation effect caused by ER blockade, involving systems such as reproduction, bone, and vasomotor. Its mechanism is consistent with the role of the drug in ER breast cancer [21].

The table further includes immunotherapy drugs and biological agents, such as Adalimumab and Recombinant Human Interferon Beta-1a, whose usage in BC treatment may be linked to the occurrence of adverse events. Additionally, some chemotherapeutic drugs like Methotrexate and Cyclophosphamide were present in the list, likely due to their known side effects.

Of particular concern are drugs with limited reported cases but exceptionally high RORs, such as Progesterone, whose ROR reached 68.82. This highlights the need for careful consideration of the safety and dosage control of these drugs in the treatment of hormone receptor-positive BC.

## 4 DISCUSSION

### 4.1 Safety Issues of Drugs Used in BC Treatment

Based on our research findings, the association between BC treatment drugs and adverse events is significant. Notably, certain medications, such as Sonidegib and sacituzumab govitecan, exhibit extremely high RORs, suggesting the need for greater caution when administering these drugs. In general, ROR was used to compare the odds of reporting drug-specific adverse events with other adverse events [22]. ROR examines only the increased risk of adverse events reporting but not the occurrence risk of adverse event. In terms of clinical applicability, ROR can efficiently identify adverse drug reaction signals that require additional monitoring by quantifying the strength of the association between drugs and adverse events, setting reasonable thresholds (usually ROR>2 and 95% CI lower limit >1, combined with the threshold of the number of reports), to assist clinical decision-making and regulatory interventions, such as adjusting medication guidelines and strengthening patient monitoring [23].

The low bioavailability of Sonidegib poses a limitation on its effective concentration and duration *in vivo*, potentially impacting its pharmacological efficacy. Additionally, resistance issues related to its antitumor activity and target specificity have been raised, suggesting that tumor cells may develop resistance to Sonidegib, further compromising its therapeutic outcome [24]. While adverse reactions associated with Sonidegib have been described, the adoption of diverse strategies is necessary to better manage this small-molecule drug and prevent treatment interruptions. These strategies may encompass optimizing dosing regimens, combining it with other agents to enhance efficacy, developing novel formulations to improve bioavailability, and conducting in-depth research into its resistance mechanisms to identify means of overcoming resistance. By implementing these measures, we can utilize Sonidegib more effectively and offer patients better therapeutic options [25].

Sacituzumab Govitecan is an advanced antibody drug conjugate (ADC) specifically designed for the treatment of HER2-negative metastatic BC (mBC) by conjugating SN-38, the active metabolite of irinotecan, to a humanized monoclonal antibody targeting Trop-2. In ongoing clinical studies, the primary grade 3 or higher adverse events observed include neutropenia (43%), anemia (9.5%), diarrhea (7.0%), and febrile neutropenia (6.3%) [26]. In studies evaluating the efficacy and tolerability of sacituzumab govitecan in patients with HER2-negative metastatic BC, the drug has demonstrated remarkable therapeutic outcomes. Notably, in the TROPiCS-02 trial, compared to the standard treatment of physician's choice, sacituzumab govitecan significantly improved the patients' progression-free survival (PFS), extending it from 4 months to 5.5 months, and also prolonged overall survival (OS), increasing it from 11.2 months to 14.4 months. It is worth noting that this improvement in efficacy was not restricted by the level of Trop-2 expression [27].

In terms of safety evaluation, the safety profile of sacituzumab govitecan is consistent with earlier study findings. While patients may experience adverse reactions such as neutropenia, diarrhea, and nausea, these symptoms can be effectively managed with appropriate supportive care measures [28]. Furthermore, patient-reported outcomes (PROs) indicate that compared to TPC, sacituzumab govitecan significantly delays the overall decline in quality of life and deterioration of physical and emotional functioning. Additionally, it mitigates the exacerbation of symptoms such as fatigue, dyspnea, and insomnia [29]. When compared to treatment of TPC the overall least square mean change from baseline for sacituzumab govitecan was markedly superior in terms of physical functioning and dyspnea, whereas it was inferior for diarrhea [30]. In the subgroup analysis of patients with metastatic triple-negative breast cancer, sacituzumab govitecan demonstrated good tolerability and manageable safety, consistent with the overall safety population. The most common treatment-emergent adverse events across all subgroups were neutropenia and diarrhea [31]. Furthermore, although some commonly used drugs such as Docetaxel and Capecitabine have relatively low RORs, their absolute number of adverse events remains high due to their widespread use.

### 4.2 Application of Precision Medicine in BC Treatment

The application of precision medicine in BC treatment primarily manifests through the utilization of molecular profiling analysis to provide personalized therapeutic regimens for patients. The classification of BC, based on the expression of hormone receptors and HER2 status in tumors, serves as the foundation for precision medicine. With advancements in technology, clinicians are now able to more meticulously consider patients' biomarker expression profiles, enabling the selection of treatment strategies that are even more tailored to their individual needs.

For instance, in HER2-positive BC patients, Trastuzumab emtansine (T-DM1), an antibody-drug conjugate that combines the anti-tumor properties targeting HER2 with the cytotoxic activity of the microtubule inhibitor DM1, has demonstrated longer progression-free survival and overall survival compared to traditional chemotherapeutic agents in clinical trials [32]. The successful application of this treatment approach showcases the potential of precision medicine in BC therapy. Moreover, with the in-depth study of the molecular mechanisms of BC, new therapeutic targets are constantly being discovered. For instance, CDK4/6 inhibitors, inhibitors of the PI3K/AKT signaling pathway, and PARP inhibitors are all novel therapies developed based on specific molecular characteristics [33]. The selection of these therapeutic methods relies on detailed molecular analysis of patients' tumor samples, embodying the core concept of precision medicine. The advancement of precision medicine also encompasses a holistic consideration of the patient's overall condition, exemplified by the application of immunotherapy. This approach transcends solely targeting the cancer itself and encompasses the modulation and support of the patient's immune system. It endeavors to achieve comprehensive, individualized treatment for patients, spanning from the microscopic to the macroscopic level.

### 4.3 Individualized Selection of Therapeutic Drugs

In the course of BC treatment, the selection of medications represents a complex yet pivotal task. Among the critical issues that cannot be overlooked is cardiotoxicity, which is particularly evident with certain chemotherapeutic agents. Notably, doxorubicin, trastuzumab, and epirubicin have been established to be associated with cardiac toxicity [34]. Additionally, drugs such as paclitaxel may also precipitate cardiac issues, including arrhythmias [34].

To mitigate the risk of cardiotoxicity, we must adopt a series of strategies. Foremost, conducting a comprehensive assessment of patients to identify potential cardiac risk factors is imperative [35, 36]. This encompasses evaluating patients' age, history of cardiac diseases, and whether they are currently using other medications or therapies that may exacerbate cardiac risks. Secondly, leveraging advanced diagnostic techniques and highly sensitive laboratory assays for early detection of cardiotoxicity is critical. These methodologies facilitate the identification of latent cardiac issues prior to symptom manifestation, enabling prompt preventive measures. Furthermore, seamless collaboration with cardiology experts within a multidisciplinary treatment team is vital. This ensures that therapeutic regimens are both efficacious and safe, optimizing care for patients with comorbid cardiovascular conditions and cancer [36, 37].

For patients who develop cardiotoxicity, adjustments or alterations to the treatment plan aimed at mitigating cardiac injury are necessary [34]. This may involve the utilization of cardioprotective agents such as beta-blockers and ACE inhibitors to alleviate the effects of cardiotoxicity [34]. The management of BC necessitates a holistic approach that not only focuses on drug efficacy but also integrates considerations of patients' overall health status, particularly cardiac health, to achieve optimal individualized treatment outcomes.

### 4.4 Monitoring and Management of Adverse Drug Reactions

By delving into the data within the FAERS, we can identify drugs with elevated risks of adverse reactions. Like many prior studies, we leverage FAERS to detect disproportionate ADR signals. This aligns with methodologies with EudraVigilance [38] or VigiBase [39], which similarly rely on healthcare provider/patient reports to generate safety signals. The innovative points of this study narrow its scope to BC therapies and their concomitant/supportive care medications. This targeted approach allows for deeper contextualization of ADRs within the clinical realities of BC treatment such as adjuvant therapy sequences, a gap in most pan-disease analyses using EudraVigilance. Employing statistical methods, such as calculating the ROR and Information Component, allows for the assessment of the strength of association between specific drugs and adverse events. Additionally, the application of machine learning technologies further enhances the prediction and analysis of drug adverse reaction patterns [40].

Comparative analysis of FAERS data sheds light on the disparities in adverse reactions among different drug groups, providing a scientific basis for clinical decision-making. By contrasting the incidence rates of adverse reactions across drug groups, we can uncover potential safety concerns within specific patient populations. The medical error reports documented in FAERS serve as invaluable resources for identifying and preventing potential medication errors. Rigorous analysis of these data reveals common error types and drugs with heightened risks, enabling the implementation of effective prevention strategies [41].

While the FAERS database offers a wealth of adverse reaction data, issues pertaining to the incompleteness and accuracy of reporting cannot be overlooked. Standardizing drug names and optimizing data quality control procedures can significantly enhance the precision and reliability of the data [42]. The low number of HER2+ and TNBC cases reflects underreporting of molecular subtype data in FAERS, as subtype documentation is not mandatory and relies on variable free-text entries. This may underestimate true subtype-specific safety signals, particularly for older therapies or in regions with less molecular testing availability.

The disparity in case counts stems from FAERS’ inherent limitations in capturing molecular subtype data, compounded by our conservative inclusion criteria to ensure accuracy. While this may underrepresent true subtype prevalence, it strengthens the validity of identified signals by focusing on well-documented cases. Future research could leverage linked datasets to validate subtype-specific risks, but within the constraints of FAERS, our approach balances specificity with exploratory signal detection.

### 4.5 Future Directions in BC Treatment

The latest advancements in BC treatment encompass molecular subtyping and personalized therapies, the development of targeted drugs, the application of immunotherapy, challenges in drug resistance, and multidisciplinary comprehensive treatment strategies. With an increasing understanding of the biological characteristics of BC, treatment approaches have shifted from traditional modalities such as surgery, radiation therapy, and chemotherapy towards personalized treatments tailored to patients' specific molecular subtypes [8, 43]. Newly developed targeted drugs, which aim at distinct molecular pathways in BC, are currently under clinical evaluation for their efficacy. Immunotherapy, by activating the patient's own immune system, offers new therapeutic avenues for patients with advanced BC [44]. Concurrently, in the face of drug resistance challenges, new clinical trials are exploring ways to identify and overcome resistance mechanisms. Additionally, BC treatment is transitioning towards multidisciplinary comprehensive treatment strategies, integrating surgery, radiation therapy, chemotherapy, targeted therapy, and immunotherapy, with the goal of enhancing treatment efficacy and improving patients' quality of life [45]. Recent studies have indicated that pyrotinib in combination with docetaxel can serve as a first-line treatment option for patients with HER2-positive metastatic BC, demonstrating acceptable safety and promising antitumor activity [46]. In mouse models, administering a drug substance (DS) of docetaxel *via* a low-dose delivery system (LDDS) enhances the differentiation of T helper cells, resulting in improved survival rates and therapeutic outcomes. From a safety standpoint, utilizing LDDS to deliver low concentrations of docetaxel directly to lymph nodes (LNs), without any additional anticancer treatments, presents a novel and promising approach for managing LN metastasis in cancer [47]. This study currently analyzes and points out the adverse conditions after treating with diverse therapeutic strategics for BC, indicating the attention of medication safety. It is considered as the innovation to identify the impact of drug-induced adverse event across BC therapies, compared to other researches. Limitation of this study is lack of the integration of potential mechanisms for adverse events in BC patients. Future studies should explore longitudinal monitoring of high-ROR drugs and integrate multi-omics data may elucidate mechanistic links between drugs and adverse events.

### 4.6 Regional Disparities and Allocation of Medical Resources

To address disparities in medical resources across regions and enhance the quality and safety of patient care, a multifaceted approach is warranted: Initially, the proficiency of healthcare providers in novel BC therapies should be elevated through remote education and online courses, enhancing their understanding and application of these treatments [48]. Subsequently, a centralized national pharmaceutical distribution system should be established to streamline drug allocation, ensuring timely availability and inventory adjustment based on regional demands [49]. Additionally, personalized treatment protocols should be advocated, empowering physicians to tailor treatment plans to individual patient characteristics [50]. Concurrently, patient access to information should be augmented through social media and online platforms, facilitating a more profound comprehension of therapeutic options and potential side effects [50]. Furthermore, inter-regional collaboration among medical institutions should be fortified to share data and best practices, thereby promoting the equitable distribution of medical resources. Ultimately, governmental and regulatory bodies must undertake regular assessments and adjustments of healthcare policies, with continuous monitoring of training requirements, pharmaceutical supply chains, and patient satisfaction indices to ensure the efficiency and equity of medical services [51].

## CONCLUSION

This comprehensive analysis of FAERS data highlights significance in drug-induced adverse event reporting across BC therapies. Targeted therapies of lapatinib or fulvestrant and endocrine agents (*e.g.*, letrozole, ribociclib) show disproportionately high AE reporting odds ratios, necessitating vigilant monitoring. HER2-positive BC treatments (trastuzumab) and TNBC agents (sonidegib) exhibit distinct risk profiles, advocating for personalized risk-benefit assessments. These findings underscore the importance of proactive risk communication, dose adjustments, and post-marketing surveillance for high-risk drugs for clinicians and regulators for risk-based monitoring protocols or patient counseling strategies. Patients and clinicians should prioritize drugs with lower RORs (*e.g.*, capecitabine) when alternatives exist. Future research necessitates ongoing attention to the prevention and management of adverse drug reactions by regulatory agencies, while simultaneously exploring more personalized and precise treatment protocols by clinicians. This work advances patient safety by providing actionable evidence for therapeutic decision-making in the oncology of BC.

## Figures and Tables

**Fig. (1):**
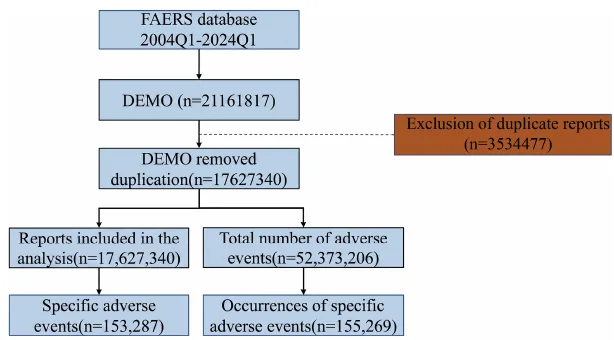
The Flow Chart of the Study.

**Fig. (2):**
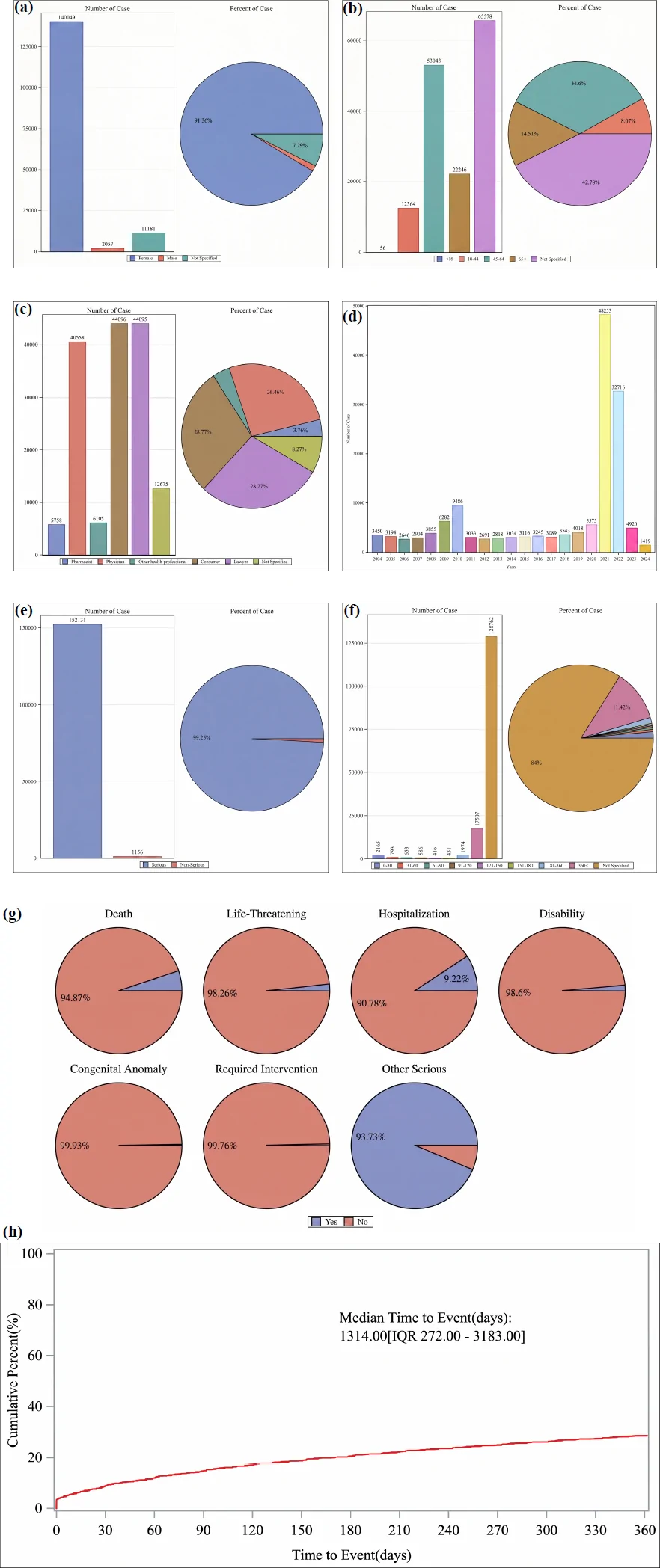
Basic Information for BC Patients with Specific Adverse Events. (**a**) Gender, (**b**) Age, (**c**) OCCP, (**d**) Get Data Year, (**e**) Serious Report, (**f**) Time of Adverse Event Occurrence, (**g**) Outcomes, (**h**) Time to Event.

**Table 1:** Chi-square Test with a 2x2.

	**Target Adverse Event Counts**	**Other Adverse Event Counts**	**Total**
Target Drug	a	b	a+b
Other Drugs	c	d	c+d
Total	a+c	b+d	N=a+b+c+d

**Note:** To illustrate the specific meanings of a, b, c, and d with a particular example, when studying the degree of association between the “target drug” and “elevated blood pressure,” a represents the number of adverse event cases of elevated blood pressure caused by the target drug; b represents the total number of adverse event cases caused by the target drug excluding those of elevated blood pressure; c represents the number of adverse event cases of elevated blood pressure caused by drugs other than the target drug; d represents the number of adverse event cases caused by drugs other than the target drug excluding those of elevated blood pressure; n denotes the total number of adverse event cases occurring in the background data included in the analysis.

**Table 2:** Reporting odds ratio (ROR) method.

**Calculation Formula**	**Threshold**
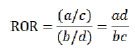	a ≥ 3, and The lower limit of the 95% confidence interval (CI) > 1
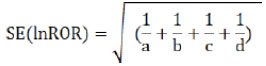	
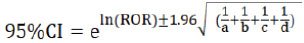	

**Table 3:** Basic patient information classification for patients with specific adverse events (breast cancer).

**Indicator**	**Number of Cases (%)**
Gender	-
Female (%)	78179 (88.40)
Male (%)	1102 (1.25)
Not Specified (%)	9152 (10.35)
Age	-
< 18 (%)	29 (0.03)
18-44 (%)	4743 (5.36)
45-64 (%)	24430 (27.63)
65 ≤ (%)	11209 (12.68)
NotSpecified (%)	48022 (54.30)
Age (Quantitative)	-
N (Missing)	40411 (48022)
Mean (SD)	57.96 (11.50)
Median (Q1, Q3)	58.00 (50.00,65.00)
Min, Max	0.00,104.00
Year of Report	-
2004 (%)	1054 (1.19)
2005 (%)	1388 (1.57)
2006 (%)	1025 (1.16)
2007 (%)	928 (1.05)
2008 (%)	2430 (2.75)
2009 (%)	3924 (4.44)
2010 (%)	5106 (5.77)
2011 (%)	1574 (1.78)
2012 (%)	1726 (1.95)
2013 (%)	1870 (2.11)
2014 (%)	1986 (2.25)
2015 (%)	2007 (2.27)
2016 (%)	1891 (2.14)
2017 (%)	1923 (2.17)
2018 (%)	2184 (2.47)
2019 (%)	2433 (2.75)
2020 (%)	3539 (4.00)
2021 (%)	38015 (42.99)
2022 (%)	10401 (11.76)
2023 (%)	2373 (2.68)
2024 (%)	656 (0.74)
Reporter	-
Consumer (%)	34606 (39.13)
Lawyer (%)	30113 (34.05)
Not Specified (%)	6432 (7.27)
Other health-professional (%)	3673 (4.15)
Pharmacist (%)	3116 (3.52)
Physician (%)	10493 (11.87)
Continent of the Occurrence Country	-
North America (%)	59499 (67.28)
Not Specified (%)	19701 (22.28)
Europe (%)	5521 (6.24)
Asia (%)	1885 (2.13)
South America (%)	1320 (1.49)
Oceania (%)	380 (0.43)
Africa (%)	127 (0.14)
Occurrence Country	-
United States of America (%)	56686 (64.10)
Canada (%)	2594 (2.93)
Japan (%)	1203 (1.36)
Germany (%)	1156 (1.31)
United Kiongdom (%)	1013 (1.15)
Continent of the Reporting Country	-
North America (%)	65708 (74.30)
Not Specified (%)	11706 (13.24)
Europe (%)	6908 (7.81)
Asia (%)	2129 (2.41)
South America (%)	1376 (1.56)
Oceania (%)	466 (0.53)
Africa (%)	140 (0.16)
Reporting Country	-
United States of America (%)	62626 (70.82)
Canada (%)	2839 (3.21)
Germany (%)	1421 (1.61)
United Kiongdom (%)	1417 (1.60)
Japan (%)	1374 (1.55)
Serious Report	-
Serious (%)	87841 (99.33)
Non-Serious (%)	592 (0.67)
Outcomes	-
Life-Threatening (%)	1218 (1.38)
Hospitalization - Initial or Prolonged (%)	7751 (8.76)
Disability (%)	903 (1.02)
Death (%)	3624 (4.10)
Congenital Anomaly (%)	9 (0.01)
Required Intervention to Prevent Permanent Impairment/Damage (%)	172 (0.19)
Other (%)	83360 (94.26)
Time of Adverse Event Occurrence - Days of Drug Use Segmentation	-
0-30 d (%)	1140 (1.29)
31-60 d (%)	409 (0.46)
61-90 d (%)	314 (0.36)
91-120 d (%)	260 (0.29)
121-150 d (%)	177 (0.20)
151-180 d (%)	212 (0.24)
181-360 d (%)	908 (1.03)
360 d < (%)	7146 (8.08)
Missing or Abnormal Values (Less Than 0) (%)	77867 (88.05)
Time of Adverse Event Occurrence - Days of Drug Use (Set Blank for Less Than 0	-
N (Missing)	10566 (77867)
Mean (SD)	1735.80 (2122.02)
Median (Q1, Q3)	957.50 (201.00,2598.00)
Min, Max	0.00,37069.0

**Table 4:** Drugs ranked by frequency of specific adverse events breast cancer associated with their use.

**Drug**	**Number(N)**	**ROR (95% CI)**
LETROZOLE	700	5.52 (5.12-5.94)
ANASTROZOLE	295	3.28 (2.92-3.68)
CAPECITABINE	286	1.09 (0.97-1.22)
TRASTUZUMAB	270	1.72 (1.52-1.93)
FULVESTRANT	249	4.19 (3.70-4.75)
RALOXIFENE	248	7.33 (6.47-8.31)
LAPATINIB	202	3.40 (2.96-3.91)
EXEMESTANE	194	5.11 (4.44-5.89)

**Table 5:** Top 10 drugs ranked by frequency of occurrence of specific adverse events in breast cancer patients.

**Drug**	**Numbers (N)**	**ROR (95% CI)**
RANITIDINE	45456	103.29 (101.90-104.68)
ESTROGENS CONJUGATED; MEDROXYPROGESTERONE	5823	278.88 (270.21-287.82)
ESTROGENS CONJUGATED	3175	54.54 (52.56-56.59)
MEDROXYPROGESTERONE	2425	31.79 (30.51-33.14)
ETANERCEPT	1582	0.68 (0.65-0.71)
INTERFERON BETA-1A	1309	1.58 (1.50-1.67)
INFLIXIMAB	1271	1.05 (0.99-1.11)
ADALIMUMAB	866	0.26 (0.25-0.28)
ESTRADIOL	818	4.77 (4.45-5.12)
LETROZOLE	700	5.52 (5.12-5.94)

**Table 6:** Basic information of patients with metastatic breast cancer.

**Indicator**	**Number of Cases (%)**
Gender	-
Female (%)	8597 (92.90)
Male (%)	66 (0.71)
Not Specified (%)	591 (6.39)
Age	-
< 18 (%)	0 (0.00)
18-44 (%)	540 (5.84)
45-64 (%)	2584 (27.92)
65 ≤ (%)	1473 (15.92)
NotSpecified (%)	4657 (50.32)
Age (Quantitative)	-
N (Missing)	4597 (4657)
Mean (SD)	58.89 (12.30)
Median (Q1, Q3)	59.00 (51.00,67.00)
Min, Max	19.00,101.00
Year of Report	-
2004 (%)	86 (0.93)
2005 (%)	111 (1.20)
2006 (%)	137 (1.48)
2007 (%)	406 (4.39)
2008 (%)	910 (9.83)
2009 (%)	602 (6.51)
2010 (%)	474 (5.12)
2011 (%)	327 (3.53)
2012 (%)	313 (3.38)
2013 (%)	284 (3.07)
2014 (%)	271 (2.93)
2015 (%)	436 (4.71)
2016 (%)	492 (5.32)
2017 (%)	352 (3.80)
2018 (%)	387 (4.18)
2019 (%)	480 (5.19)
2020 (%)	662 (7.15)
2021 (%)	831 (8.98)
2022 (%)	664 (7.18)
2023 (%)	861 (9.30)
2024 (%)	168 (1.82)
Reporter	-
Consumer (%)	3257 (35.20)
Lawyer (%)	1030 (11.13)
Not Specified (%)	291 (3.14)
Other health-professional (%)	936 (10.11)
Pharmacist (%)	898 (9.70)
Physician (%)	2842 (30.71)
Continent of the Occurrence Country	-
North America (%)	3524 (38.08)
Not Specified (%)	2800 (30.26)
Europe (%)	2062 (22.28)
Asia (%)	380 (4.11)
South America (%)	276 (2.98)
Oceania (%)	157 (1.70)
Africa (%)	55 (0.59)
Occurrence Country	-
United States of America (%)	1994 (21.55)
Canada (%)	751 (8.12)
Germany (%)	656 (7.09)
United Kiongdom (%)	271 (2.93)
Netherlands (%)	163 (1.76)
Continent of the Reporting Country	-
North America (%)	5562 (60.10)
Not Specified (%)	2278 (24.62)
Europe (%)	499 (5.39)
Asia (%)	450 (4.86)
South America (%)	245 (2.65)
Oceania (%)	165 (1.78)
Africa (%)	55 (0.59)
Reporting Country	-
United States of America (%)	4649 (50.24)
Canada (%)	850 (9.19)
Germany (%)	693 (7.49)
United Kiongdom (%)	375 (4.05)
Australia (%)	159 (1.72)
Serious Report	-
Serious (%)	9013 (97.40)
Non-Serious (%)	241 (2.60)
Outcomes	-
Life-Threatening (%)	290 (3.13)
Hospitalization - Initial or Prolonged (%)	1909 (20.63)
Disability (%)	134 (1.45)
Death (%)	2545 (27.50)
Congenital Anomaly (%)	4 (0.04)
Required Intervention to Prevent Permanent Impairment/Damage (%)	11 (0.12)
Other (%)	7476 (80.79)
Time of Adverse Event Occurrence - Days of Drug Use Segmentation	-
0-30 d (%)	472 (5.10)
31-60 d (%)	132 (1.43)
61-90 d (%)	114 (1.23)
91-120 d (%)	93 (1.00)
121-150 d (%)	51 (0.55)
151-180 d (%)	51 (0.55)
181-360 d (%)	217 (2.34)
360 d < (%)	1025 (11.08)
Missing or Abnormal Values (Less Than 0) (%)	7099 (76.71)
Time of Adverse Event Occurrence - Days of Drug Use (Set Blank for Less Than 0	-
N (Missing)	2155 (7099)
Mean (SD)	1053.23 (1703.55)
Median (Q1, Q3)	306.00 (43.00,1402.00)
Min, Max	0.00,22280.0

**Table 7:** Frequency of specific adverse events in metastatic breast cancer by medication.

**Drug**	**Number (N)**	**ROR (95% CI)**
ESTROGENS CONJUGATED; MEDROXYPROGESTERONE	1433	544.89 (514.07-577.56)
LETROZOLE	668	53.84 (49.75-58.27)
ESTROGENS CONJUGATED	663	105.74 (97.65-114.51)
RIBOCICLIB	610	55.07 (50.72-59.81)
TRASTUZUMAB	378	23.92 (21.58-26.52)
CAPECITABINE	319	11.97 (10.71-13.39)
DENOSUMAB	263	4.85 (4.29-5.48)
ZOLEDRONIC ACID	260	5.62 (4.97-6.36)
EVEROLIMUS	222	9.84 (8.61-11.24)
LAPATINIB	215	35.40 (30.91-40.55)
ANASTROZOLE	204	22.07 (19.21-25.36)
PACLITAXEL	185	10.19 (8.81-11.79)
EXEMESTANE	137	34.88 (29.45-41.32)
ADALIMUMAB	84	0.25 (0.20-0.30)
TAMOXIFEN	75	28.78 (22.92-36.15)
DOCETAXEL	73	2.37 (1.88-2.99)
DOXORUBICIN	72	4.30 (3.41-5.43)
CYCLOPHOSPHAMIDE	60	4.14 (3.21-5.34)
GEMCITABINE	37	2.70 (1.96-3.73)
NERATINIB	37	30.34 (21.95-41.93)
PERTUZUMAB	32	10.65 (7.53-15.08)
EPIRUBICIN	25	7.55 (5.10-11.19)
ESTRADIOL	25	1.38 (0.93-2.04)

**Table 8:** Clinical characteristics of HER2-positive BC patients.

**Indicator**	**Number of Cases (%)**
Gender	-
Female (%)	251 (78.93)
Male (%)	7 (2.20)
Not Specified (%)	60 (18.87)
Age	-
< 18 (%)	1 (0.31)
18-44 (%)	45 (14.15)
45-64 (%)	109 (34.28)
65 ≤ (%)	24 (7.55)
NotSpecified (%)	139 (43.71)
Age (Quantitative)	-
N (Missing)	179 (139)
Mean (SD)	52.17 (11.90)
Median (Q1, Q3)	54.00 (44.00,61.00)
Min, Max	17.00,77.00
Year of Report	-
2004 (%)	2 (0.63)
2005 (%)	8 (2.52)
2006 (%)	11 (3.46)
2007 (%)	20 (6.29)
2008 (%)	12 (3.77)
2009 (%)	15 (4.72)
2010 (%)	45 (14.15)
2011 (%)	23 (7.23)
2012 (%)	23 (7.23)
2013 (%)	47 (14.78)
2014 (%)	45 (14.15)
2015 (%)	55 (17.30)
2016 (%)	12 (3.77)
2017 (%)	-
2018 (%)	61 (19.18)
2019 (%)	3 (0.94)
2020 (%)	6 (1.89)
2021 (%)	28 (8.81)
2022 (%)	88 (27.67)
2023 (%)	132 (41.51)
2024 (%)	-
Reporter	136 (42.77)
Consumer (%)	104 (32.70)
Lawyer (%)	23 (7.23)
Not Specified (%)	19 (5.97)
Other health-professional (%)	19 (5.97)
Pharmacist (%)	15 (4.72)
Physician (%)	2 (0.63)
Occurrence Country	-
United States of America (%)	85 (26.73)
United Kiongdom (%)	34 (10.69)
Canada (%)	18 (5.66)
Portugal (%)	16 (5.03)
Australia (%)	15 (4.72)
Continent of the Reporting Country	-
North America (%)	140 (44.03)
Not Specified (%)	113 (35.53)
Europe (%)	23 (7.23)
Asia (%)	18 (5.66)
South America (%)	15 (4.72)
Oceania (%)	7 (2.20)
Africa (%)	2 (0.63)
Reporting Country	-
United States of America (%)	93 (29.25)
United Kiongdom (%)	35 (11.01)
Canada (%)	18 (5.66)
Germany (%)	17 (5.35)
Australia (%)	15 (4.72)
Serious Report	-
Serious (%)	307 (96.54)
Non-Serious (%)	11 (3.46)
Outcomes	-
Life-Threatening (%)	15 (4.72)
Hospitalization - Initial or Prolonged (%)	44 (13.84)
Disability (%)	10 (3.14)
Death (%)	34 (10.69)
Congenital Anomaly (%)	0 (0.00)
Required Intervention to Prevent Permanent Impairment/Damage (%)	5 (1.57)
Other (%)	280 (88.05)
Time of Adverse Event Occurrence - Days of Drug Use Segmentation	-
0-30 d (%)	8 (2.52)
31-60 d (%)	2 (0.63)
61-90 d (%)	1 (0.31)
91-120 d (%)	1 (0.31)
121-150 d (%)	0 (0.00)
151-180 d (%)	1 (0.31)
181-360 d (%)	5 (1.57)
360d < (%)	18 (5.66)
Missing or Abnormal Values (Less Than 0) (%)	282 (88.68)
Time of Adverse Event Occurrence - Days of Drug Use (Set Blank for Less Than 0	-
N(Missing)	36 (282)
Mean (SD)	1002.31 (1421.02)
Median (Q1, Q3)	342.50 (40.00,1355.00)
Min, Max	0.00,5847.00

**Table 9:** Top 9 drugs ranked by frequency of occurrence of specific adverse events in HER2-positive breast cancer patients.

**Drug**	**Number (N)**	**ROR (95% CI)**
TRASTUZUMAB	90	221.11 (173.23-282.24)
FINGOLIMOD	13	7.90 (4.53-13.76)
RANITIDINE	11	3.23 (1.77-5.89)
LEVONORGESTREL	11	4.23 (2.32-7.72)
OCRELIZUMAB	9	10.72 (5.52-20.79)
INTERFERON BETA-1A	9	3.07 (1.58-5.95)
ESTRADIOL	8	13.11 (6.50-26.45)
DOCETAXEL	8	7.70 (3.82-15.54)
LETROZOLE	8	17.71 (8.78-35.73)

**Table 10:** Basic information of triple-negative BC patients.

**Indicator**	**Number of Cases (%)**
Gender	-
Female (%)	280 (92.11)
Not Specified (%)	24 (7.89)
Age	-
< 18 (%)	0 (0.00)
18-44 (%)	57 (18.75)
45-64 (%)	97 (31.91)
65 ≤ (%)	36 (11.84)
NotSpecified (%)	114 (37.50)
Age (Quantitative)	-
N (Missing)	190 (114)
Mean (SD)	52.48 (13.49)
Median (Q1, Q3)	52.00 (43.00,61.00)
Min, Max	23.00,87.00
Year of Report	-
2015 (%)	8 (2.63)
2016 (%)	10 (3.29)
2017 (%)	13 (4.28)
2018 (%)	13 (4.28)
2019 (%)	43 (14.14)
2020 (%)	41 (13.49)
2021 (%)	52 (17.11)
2022 (%)	58 (19.08)
2023 (%)	52 (17.11)
2024 (%)	14 (4.61)
Reporter	-
Consumer (%)	112 (36.84)
Lawyer (%)	6 (1.97)
Not Specified (%)	7 (2.30)
Other health-professional (%)	18 (5.92)
Pharmacist (%)	45 (14.80)
Physician (%)	116 (38.16)
Continent of the Occurrence Country	-
North America (%)	143 (47.04)
Europe (%)	84 (27.63)
Asia (%)	33 (10.86)
Oceania (%)	24 (7.89)
Not Specified (%)	14 (4.61)
South America (%)	6 (1.97)
Occurrence Country	-
United States of America (%)	134 (44.08)
Germany (%)	31 (10.20)
Australia (%)	24 (7.89)
Japan (%)	23 (7.57)
France (%)	18 (5.92)
Continent of the Reporting Country	-
North America (%)	159 (52.30)
Europe (%)	86 (28.29)
Asia (%)	32 (10.53)
Oceania (%)	15 (4.93)
Not Specified (%)	7 (2.30)
South America (%)	5 (1.64)
Reporting Country	-
United States of America (%)	150 (49.34)
Germany (%)	30 (9.87)
Japan (%)	22 (7.24)
France (%)	19 (6.25)
Australia (%)	15 (4.93)
Serious Report	-
Serious (%)	303 (99.67)
Non-Serious (%)	1 (0.33)
Outcomes	-
Life-Threatening (%)	20 (6.58)
Hospitalization - Initial or Prolonged (%)	45 (14.80)
Disability (%)	13 (4.28)
Death (%)	33 (10.86)
Congenital Anomaly (%)	0 (0.00)
Required Intervention to Prevent Permanent Impairment/Damage (%)	3 (0.99)
Other (%)	268(88.16)
Time of Adverse Event Occurrence - Days of Drug Use Segmentation	-
0-30 d (%)	16 (5.26)
31-60 d (%)	3 (0.99)
61-90 d (%)	8 (2.63)
91-120 d (%)	2 (0.66)
121-150 d (%)	2 (0.66)
151-180 d (%)	1 (0.33)
181-360 d (%)	7 (2.30)
360d < (%)	16 (5.26)
Missing or Abnormal Values (Less Than 0) (%)	249 (81.91)
Time of Adverse Event Occurrence - Days of Drug Use (Set Blank for Less Than 0	-
N (Missing)	55 (249)
Mean (SD)	521.80 (908.37)
Median (Q1, Q3)	101.00 (17.00,612.00)
Min, Max	0.00,3931.00

**Table 11:** Top 9 drugs ranked by frequency of occurrence of specific adverse events in triple-negative breast cancer patients.

**Drug**	**Number (N)**	**ROR (95% CI)**
ATEZOLIZUMAB	22	85.05 (55.11-131.26)
RANITIDINE	17	5.33 (3.27-8.70)
SACITUZUMAB GOVITECAN	15	286.78 (170.59-482.10)
ADALIMUMAB	13	1.20 (0.69-2.08)
DOCETAXEL	11	11.20 (6.14-20.45)
CAPECITABINE	10	11.38 (6.06-21.38)
DENOSUMAB	9	5.06 (2.61-9.81)
SONIDEGIB	8	885.20 (437.84-1789.68)
PACLITAXEL	8	13.49 (6.68-27.22)

**Table 12:** Basic clinical information of hormone receptor-positive breast cancer patients.

**Indicator**	**Number of Cases (%)**
Gender	-
Female (%)	258 (87.16)
Male (%)	17 (5.74)
Not Specified (%)	21 (7.09)
Age	-
< 18 (%)	0 (0.00)
18-44 (%)	31 (10.47)
45-64 (%)	103 (34.80)
65 ≤ (%)	56 (18.92)
NotSpecified (%)	106 (35.81)
Age (Quantitative)	-
N (Missing)	190 (106)
Mean (SD)	56.59 (14.52)
Median (Q1, Q3)	56.00 (45.00,67.00)
Min, Max	18.00,86.00
Year of Report	-
2012 (%)	2 (0.68)
2013 (%)	5 (1.69)
2014 (%)	11 (3.72)
2015 (%)	21 (7.09)
2016 (%)	9 (3.04)
2017 (%)	10 (3.38)
2018 (%)	26 (8.78)
2019 (%)	35 (11.82)
2020 (%)	40 (13.51)
2021 (%)	45 (15.20)
2022 (%)	29 (9.80)
2023 (%)	54 (18.24)
2024 (%)	9 (3.04)
Reporter	-
Consumer (%)	104 (35.14)
Lawyer (%)	6 (2.03)
Not Specified (%)	7 (2.36)
Other health-professional (%)	30 (10.14)
Pharmacist (%)	44 (14.86)
Physician (%)	105 (35.47)
Continent of the Occurrence Country	-
North America (%)	139 (46.96)
Europe (%)	95 (32.09)
Not Specified (%)	18 (6.08)
Oceania (%)	16 (5.41)
South America (%)	15 (5.07)
Asia (%)	11 (3.72)
Africa (%)	2 (0.68)
Occurrence Country	-
United States of America (%)	118 (39.86)
Germany (%)	23 (7.77)
Canada (%)	21 (7.09)
United Kiongdom (%)	20 (6.76)
Australia (%)	16 (5.41)
Continent of the Reporting Country	-
North America (%)	156 (52.70)
Europe (%)	93 (31.42)
Oceania (%)	16 (5.41)
South America (%)	15 (5.07)
Asia (%)	11 (3.72)
Not Specified (%)	3 (1.01)
Africa (%)	2 (0.68)
Reporting Country	-
United States of America (%)	135 (45.61)
Germany (%)	24 (8.11)
Canada (%)	21 (7.09)
United Kingdom (%)	19 (6.42)
Australia (%)	16 (5.41)
Serious Report	-
Serious (%)	289 (97.64)
Non-Serious (%)	7 (2.36)
Outcomes	-
Life-Threatening (%)	22 (7.43)
Hospitalization - Initial or Prolonged (%)	74 (25.00)
Disability (%)	13 (4.39)
Death (%)	2 (0.68)
Congenital Anomaly (%)	1 (0.34)
Required Intervention to Prevent Permanent Impairment/Damage (%)	4 (1.35)
Other (%)	251 (84.80)
Time of Adverse Event Occurrence - Days of Drug Use Segmentation	-
0-30 d (%)	5 (1.69)
31-60 d (%)	2 (0.68)
61-90 d (%)	2 (0.68)
91-120 d (%)	4 (1.35)
121-150 d (%)	0 (0.00)
151-180 d (%)	1 (0.34)
181-360 d (%)	2 (0.68)
360 d < (%)	35 (11.82)
Missing or Abnormal Values (Less Than 0) (%)	245 (82.77)
Time of Adverse Event Occurrence - Days of Drug Use (Set Blank for Less Than 0	-
N(Missing)	51 (245)
Mean (SD)	1233.10 (1452.18)
Median (Q1, Q3)	892.00 (118.00,1313.00)
Min, Max	0.00,6396.00

**Table 13:** Top 11 drugs associated with specific adverse events in hormone receptor-positive breast cancer ranked by frequency.

**Drug**	**Number (N)**	**ROR (95% CI)**
LEVONORGESTREL	29	12.82 (8.74-18.80)
LETROZOLE	20	49.73 (31.59-78.30)
ESTRADIOL	15	27.12 (16.14-45.60)
ANASTROZOLE	10	34.12 (18.16-64.09)
RANITIDINE	10	3.15 (1.68-5.91)
ADALIMUMAB	9	0.84 (0.43-1.63)
INTERFERON BETA-1A	9	3.30 (1.70-6.41)
EVEROLIMUS	8	11.10 (5.50-22.41)
OCRELIZUMAB	7	8.91 (4.21-18.86)
FULVESTRANT	7	35.76 (16.89-75.68)
TAMSULOSIN	7	32.66 (15.43-69.12)

## Data Availability

The datasets generated or analyzed during this study are available from the corresponding author on reasonable request.

## LIST OF ABBREVIATIONS

ADC: Antibody-drug Conjugate
BC: Breast Cancer
DS: Drug Substance
FAERS: FDA's Adverse Event Reporting System
HER2: Human Epidermal Growth Factor Receptor 2
HLGTs: Higher-level Group Terms
ICIs: Immune Checkpoint Inhibitors
LDDS: Low-dose Delivery System
LNs: Lymph Nodes
NER: Neutrophil-to-eosinophil Ratio
OS: Overall Survival
PFS: Progression-free Survival
PROs: Patient-reported Outcomes
PTs: Preferred Terms
ROR: Reporting Odds Ratio
SAEs: Specific Adverse Events
SMQs: Standardized medDRA Queries
SOC: System Organ Class
TNBC: Triple-negative Breast Cancer
