# Supplementary material for: An Updated Comprehensive Pharmacovigilance Study of Drug- Induced Breast Cancer Based on FDA Adverse Event Reporting System Data
Source: Curr Med Chem. 2026 Jan 7;33(24):4955–82. doi: 10.2174/0109298673376219251029174252 (PMC13688416; doi:10.2174/0109298673376219251029174252)
Supplement: Supplementary file 1 [file CMC-33-24-4955_SD1.pdf]

Supplementary Material

An Updated Comprehensive Pharmacovigilance Study of Drug-Induced Breast Cancer Based on FDA Adverse Event Reporting System Data

Ziran Qiu[1], Xinyu Liu[2], Wenqing Cao[1], Rui Li[1], Jun Yang[1], Chengyu Wang[1], Zhong Li[1], Xiaoqin Yao[1], Yuan Chen[1], Chunhua Ye[1], Shanzheng Chen[1] and Na Jin[1,*]

[1]*Department of Breast and Thyroid Surgery, Loudi Central Hospital, Loudi, 417000, China;* [2]*Department of Otolaryngology Head and Neck Surgery, Loudi Central Hospital, Loudi, 417000, China*

Table S1. The PT Terms Related to Specific Adverse Events.

Coding	Preferred Term	Numbers
10006187	Breast cancer	88433
10057654	Breast cancer female	24921
10055113	Breast cancer metastatic	9254
10006199	Breast cancer stage I	9017
10006200	Breast cancer stage II	7565
10006201	Breast cancer stage III	4636
10006202	Breast cancer stage IV	2713
10006198	Breast cancer recurrent	2617
10073095	Invasive ductal breast carcinoma	2310
10006189	Breast cancer in situ	1267
10061020	Breast cancer male	461
10073096	Invasive lobular breast carcinoma	430
10075713	Invasive breast carcinoma	336
10065430	HER2 positive breast cancer	318
10075566	Triple negative breast cancer	304
10083234	Hormone receptor positive breast cancer	296
10085481	Hormone receptor positive HER2 negative breast cancer	143
10021980	Inflammatory carcinoma of the breast	138
10073100	Metaplastic breast carcinoma	37
10083232	HER2 negative breast cancer	28
10021979	Inflammatory carcinoma of breast stage IV	12
10076935	Hormone refractory breast cancer	10
10083233	Triple positive breast cancer	8
10021978	Inflammatory carcinoma of breast stage III	8
10085561	Hormone receptor negative HER2 positive breast cancer	4
10021977	Inflammatory carcinoma of breast recurrent	2
10088985	Secretory breast carcinoma	1

Table S2. Top 10-50 Drugs Ranked by Frequency of Occurrence of Specific Adverse Events in Breast Cancer Patients.

Drug	Numbers (N)	ROR (95%CI)
NATALIZUMAB	566	0.78 (0.72-0.84)
DENOSUMAB	561	1.06 (0.97-1.15)
FINGOLIMOD	470	0.99 (0.90-1.08)
LEVONORGESTREL	469	0.63 (0.57-0.69)
METHOTREXATE	441	0.55 (0.50-0.60)
FUMARIC ACID	440	0.87 (0.80-0.96)
ZOLEDRONIC ACID	431	0.95 (0.87-1.05)
RIBOCICLIB	392	3.46 (3.13-3.82)
LENALIDOMIDE	384	0.33 (0.29-0.36)
TERIPARATIDE	384	0.62 (0.56-0.68)
SECUKINUMAB	368	0.56 (0.50-0.62)
EVEROLIMUS	352	1.60 (1.44-1.78)
RITUXIMAB	337	0.48 (0.44-0.54)
ABATACEPT	334	0.83 (0.74-0.92)
ALENDRONIC ACID	330	0.63 (0.56-0.70)
OCRELIZUMAB	326	1.36 (1.22-1.52)
USTEKINUMAB	310	1.19 (1.07-1.33)
GOLIMUMAB	303	1.67 (1.49-1.87)
CYCLOPHOSPHAMIDE	301	2.17 (1.94-2.43)
ANASTROZOLE	295	3.28 (2.92-3.68)
CAPECITABINE	286	1.09 (0.97-1.22)
TRASTUZUMAB	270	1.72 (1.52-1.93)
TOCILIZUMAB	260	0.69 (0.61-0.78)
CERTOLIZUMAB PEGOL	257	0.74 (0.65-0.83)
FULVESTRANT	249	4.19 (3.70-4.75)
INSULIN GLARGINE	248	0.65 (0.57-0.74)
RALOXIFENE	248	7.33 (6.47-8.31)
CLOZAPINE	246	0.58 (0.51-0.66)
OMALIZUMAB	238	0.71 (0.63-0.81)
APIXABAN	233	0.57 (0.50-0.65)
TOFACITINIB	232	0.37 (0.32-0.42)
VALSARTAN	229	1.41 (1.24-1.60)
ESOMEPRAZOLE	203	0.46 (0.40-0.53)
LAPATINIB	202	3.40 (2.96-3.91)
INSULIN LISPRO	201	0.60 (0.52-0.68)
EXEMESTANE	194	5.11 (4.44-5.89)

APREMILAST	184	0.43 (0.38-0.50)
PALBOCICLIB	181	0.45 (0.38-0.52)
OXYBATE SODIUM	173	0.49 (0.42-0.57)
ESTRADIOL; NORETHISTERONE	172	8.81 (7.58-10.25)

Table S3. Top 10-50 Drugs Ranked by Frequency of Occurrence of Specific Adverse Events in HER2-Positive Breast Cancer Patients.

Drug	Number (N)	ROR (95%CI)
DOXORUBICIN	7	12.35 (5.84-26.12)
CAPECITABINE	7	7.53 (3.56-15.93)
LAPATINIB	7	33.31 (15.75-70.46)
PERTUZUMAB	6	58.96 (26.28-132.26)
TRASTUZUMAB EMTANSINE	6	82.45 (36.75-184.98)
ANASTROZOLE	5	15.58 (6.44-37.71)
FULVESTRANT	5	23.58 (9.75-57.05)
PACLITAXEL	5	7.97 (3.29-19.29)
DENOSUMAB	4	2.11 (0.79-5.66)
CYCLOPHOSPHAMIDE	4	8.08 (3.01-21.66)
CARBOPLATIN	4	5.12 (1.91-13.72)
NATALIZUMAB	4	1.54 (0.57-4.12)
ADALIMUMAB	3	0.25 (0.08-0.79)
DEXAMETHASONE	3	4.14 (1.33-12.90)
RITUXIMAB	3	1.21 (0.39-3.77)
TOCILIZUMAB	3	2.22 (0.71-6.92)
ETANERCEPT	3	0.35 (0.11-1.11)
EXEMESTANE	3	21.98 (7.05-68.53)
PALBOCICLIB	3	2.07 (0.66-6.45)
ZOLEDRONIC ACID	3	1.85 (0.59-5.77)
ALEMTUZUMAB	2	4.65 (1.16-18.67)
ALENDRONIC ACID	2	1.06 (0.26-4.27)
ALPELISIB	2	14.56 (3.62-58.47)
OLAPARIB	2	11.39 (2.84-45.75)
LENALIDOMIDE	2	0.47 (0.12-1.90)
RUXOLITINIB	2	1.80 (0.45-7.23)
CLOZAPINE	2	1.32 (0.33-5.31)
NERATINIB	2	47.60 (11.85-191.20)
ETHINYLESTRADIOL; ETONOGESTREL	2	4.35 (1.08-17.47)
TAMOXIFEN	2	22.19 (5.52-89.12)

USTEKINUMAB	2	2.15 (0.53-8.62)
FUMARIC ACID	2	1.11 (0.28-4.45)
INFLIXIMAB	2	0.46 (0.11-1.83)
OXYBATE SODIUM	2	1.57 (0.39-6.32)
ABALOPARATIDE	1	3.88 (0.54-27.62)
OLANZAPINE	1	0.89 (0.13-6.35)
OXALIPLATIN	1	1.58 (0.22-11.26)
EPIRUBICIN	1	8.79 (1.23-62.59)
FAMPRIDINE	1	0.94 (0.13-6.69)
TRASTUZUMAB DERUXTECAN	1	12.30 (1.73-87.57)
FILGRASTIM	1	4.51 (0.63-32.13)

Table S4. Top 10-50 Drugs Ranked by Frequency of Occurrence of Specific Adverse Events in Triple-Negative Breast Cancer Patients.

Drug	Number (N)	ROR (95%CI)
ATEZOLIZUMAB	22	85.05 (55.11-131.26)
RANITIDINE	17	5.33 (3.27-8.70)
SACITUZUMAB GOVITECAN	15	286.78 (170.59-482.10)
ADALIMUMAB	13	1.20 (0.69-2.08)
DOCETAXEL	11	11.20 (6.14-20.45)
CAPECITABINE	10	11.38 (6.06-21.38)
DENOSUMAB	9	5.06 (2.61-9.81)
SONIDEGIB	8	885.20 (437.84-1789.68)
PACLITAXEL	8	13.49 (6.68-27.22)
METHOTREXATE	7	2.57 (1.21-5.44)
CARBOPLATIN	7	9.47 (4.47-20.03)
FUMARIC ACID	7	4.12 (1.95-8.73)
FULVESTRANT	5	24.68 (10.20-59.74)
DIROXIMEL FUMARATE	5	35.46 (14.65-85.83)
CYCLOPHOSPHAMIDE	5	10.61 (4.38-25.67)
ETANERCEPT	5	0.62 (0.26-1.51)
LEVONORGESTREL	5	1.97 (0.82-4.78)
OLAPARIB	4	24.00 (8.95-64.36)
ESTRADIOL	4	6.77 (2.53-18.17)
LETROZOLE	4	9.15 (3.41-24.54)
RISANKIZUMAB	4	7.27 (2.71-19.51)
RUXOLITINIB	4	3.79 (1.41-10.17)
NATALIZUMAB	4	1.61 (0.60-4.31)

PALBOCICLIB	4	2.90 (1.08-7.77)
PEMBROLIZUMAB	3	4.25 (1.36-13.24)
CERTOLIZUMAB PEGOL	3	2.52 (0.81-7.86)
USTEKINUMAB	3	3.38 (1.08-10.54)
ALEMTUZUMAB	2	4.86 (1.21-19.54)
OMALIZUMAB	2	1.74 (0.43-7.01)
OCRELIZUMAB	2	2.44 (0.61-9.79)
OZANIMOD	2	26.35 (6.56-105.84)
BEVACIZUMAB	2	1.74 (0.43-6.98)
DAPAGLIFLOZIN	2	6.90 (1.72-27.73)
DEXAMETHASONE	2	2.88 (0.72-11.56)
DUPILUMAB	2	0.62 (0.15-2.48)
DOXORUBICIN	2	3.63 (0.90-14.59)
GOLIMUMAB	2	3.22 (0.80-12.92)
LENALIDOMIDE	2	0.49 (0.12-1.99)
INSULIN LISPRO	2	1.73 (0.43-6.97)
RITUXIMAB	2	0.84 (0.21-3.37)
LORCASERIN	2	55.21 (13.74-221.84)
LOSARTAN	2	7.08 (1.76-28.45)
MONTELUKAST	2	3.73 (0.93-15.00)
NIVOLUMAB	2	2.11 (0.53-8.49)
TRASTUZUMAB	2	3.71 (0.92-14.89)
RIBOCICLIB	2	5.12 (1.28-20.58)
SEMAGLUTIDE	2	4.42 (1.10-17.76)
SECUKINUMAB	2	0.88 (0.22-3.55)
ECULIZUMAB	2	2.29 (0.57-9.18)
IXEKIZUMAB	2	6.75 (1.68-27.10)

Table S5. Top 12-50 Drugs Associated with Specific Adverse Events in Hormone Receptor-Positive Breast Cancer Ranked by Frequency.

Drug	Number (N)	ROR (95%CI)
METHOTREXATE	6	2.26 (1.01-5.06)
ESTROGENS CONJUGATED	6	27.87 (12.42-62.56)
RIBOCICLIB	6	16.01 (7.13-35.92)
SPIRONOLACTONE	5	34.43 (14.22-83.35)
TRASTUZUMAB	5	9.62 (3.97-23.28)
FUMARIC ACID	5	3.01 (1.24-7.28)
FINASTERIDE	4	10.54 (3.93-28.28)

CAPECITABINE	4	4.58 (1.71-12.30)
LEVOTHYROXINE	4	3.30 (1.23-8.84)
ABATACEPT	3	2.24 (0.72-6.98)
ALENDRONIC ACID	3	1.72 (0.55-5.37)
ATORVASTATIN	3	2.27 (0.73-7.08)
DENOSUMAB	3	1.70 (0.54-5.29)
METFORMIN; SITAGLIPTIN	3	21.31 (6.83-66.47)
FLUOROURACIL	3	8.91 (2.86-27.78)
CYCLOPHOSPHAMIDE	3	6.49 (2.08-20.25)
PROGESTERONE	3	68.82 (22.06-214.66)
NATALIZUMAB	3	1.24 (0.40-3.85)
CERTOLIZUMAB PEGOL	3	2.59 (0.83-8.07)
SECUKINUMAB	3	1.37 (0.44-4.26)
ACETYLSALICYLIC ACID	3	4.18 (1.34-13.05)
ZOLEDRONIC ACID	3	1.99 (0.64-6.20)
METFORMIN	2	1.58 (0.39-6.36)
DIROXIMEL FUMARATE	2	14.42 (3.59-57.95)
GEMCITABINE	2	4.58 (1.14-18.39)
RALOXIFENE	2	17.55 (4.37-70.51)
NIRAPARIB	2	2.70 (0.67-10.86)
PEMBROLIZUMAB	2	2.90 (0.72-11.65)
PAMIDRONIC ACID	2	6.27 (1.56-25.17)
PEGINTERFERON BETA-1A	2	11.56 (2.88-46.46)
ETANERCEPT	2	0.25 (0.06-1.02)
ETONOGESTREL	2	2.29 (0.57-9.19)
EXEMESTANE	2	15.70 (3.91-63.08)
PANTOPRAZOLE	2	3.50 (0.87-14.08)
ELEXACAFTOR; IVACAFTOR; TEZACAFTOR	1	6.93 (0.97-49.34)
ALPELISIB	1	7.80 (1.09-55.53)
APIXABAN	1	0.73 (0.10-5.21)
BUPROPION; NALTREXONE	1	9.97 (1.40-71.00)
OBETICHOLIC ACID	1	10.25 (1.44-72.99)
